# Comparable long-term efficacy, as assessed by patient-reported outcomes, safety and pharmacokinetics, of CT-P13 and reference infliximab in patients with ankylosing spondylitis: 54-week results from the randomized, parallel-group PLANETAS study

**DOI:** 10.1186/s13075-016-0930-4

**Published:** 2016-01-20

**Authors:** Won Park, Dae Hyun Yoo, Janusz Jaworski, Jan Brzezicki, Andriy Gnylorybov, Vladimir Kadinov, Irmgadt Goecke Sariego, Carlos Abud-Mendoza, William Jose Otero Escalante, Seong Wook Kang, Daina Andersone, Francisco Blanco, Seung Suh Hong, Sun Hee Lee, Jürgen Braun

**Affiliations:** Inha University Hospital, Incheon, Republic of Korea; Hanyang University Hospital, Seoul, Republic of Korea; Linea Corporis, Warszawa, Poland; Wojewodzki Szpital Zespolony w Elblagu, Elblag, Poland; Institute of Urgent and Recovery Surgery, Donetsk, Ukraine; University Hospital St. Marina, Varna, Bulgaria; Prosalud y Cia Ltda, Santiago, Chile; Hospital Central Dr. Ignacio Morones Prieto, San Luis Potosí, Mexico; Servimed Empresa Unipersonal, Bucaramanga, Colombia; Chungnam National University Hospital, Daejeon, Republic of Korea; P. Stradina Clinical University Hospital, Riga, Latvia; Hospital Universitario a Coruña, A Coruña, Spain; CELLTRION, Incheon, Republic of Korea; Rheumazentrum Ruhrgebiet, Herne, Germany

**Keywords:** Biosimilar, CT-P13, Infliximab, Ankylosing spondylitis, Efficacy, Immunogenicity, Pharmacokinetics, Safety, ASAS, Clinical trial

## Abstract

**Background:**

CT-P13 (Remsima®, Inflectra®) is a biosimilar of the infliximab reference product (RP; Remicade®) and is approved in Europe and elsewhere, mostly for the same indications as RP. The aim of this study was to compare the 54-week efficacy, immunogenicity, pharmacokinetics (PK) and safety of CT-P13 with RP in patients with ankylosing spondylitis (AS), with a focus on patient-reported outcomes (PROs).

**Methods:**

This was a multinational, double-blind, parallel-group study in patients with active AS. Participants were randomized (1:1) to receive CT-P13 (5 mg/kg) or RP (5 mg/kg) at weeks 0, 2, 6 and then every 8 weeks up to week 54. To assess responses, standardized assessment tools were used with an intention-to-treat analysis of observed data. Anti-drug antibodies (ADAs), PK parameters, and safety outcomes were also assessed.

**Results:**

Of 250 randomized patients (n = 125 per group), 210 (84.0 %) completed 54 weeks of treatment, with similar completion rates between groups. At week 54, Assessment of Spondylo Arthritis international Society (ASAS)20 response, ASAS40 response and ASAS partial remission were comparable between treatment groups. Changes from baseline in PROs such as mean Bath Ankylosing Spondylitis Disease Activity Index (BASDAI; CT-P13 −3.1 versus RP −2.8), Bath Ankylosing Spondylitis Functional Index (BASFI; −2.9 versus –2.7), and Short Form Health Survey (SF-36) scores (9.26 versus 10.13 for physical component summary; 7.30 versus 6.54 for mental component summary) were similar between treatment groups. At 54 weeks, 19.5 % and 23.0 % of patients receiving CT-P13 and RP, respectively, had ADAs. All observed PK parameters of CT-P13 and RP, including maximum and minimum serum concentrations, were similar through 54 weeks. The influence of ADAs on PK was similar in the two treatment groups. Most adverse events were mild or moderate in severity. There was no notable difference between treatment groups in the incidence of adverse events, serious adverse events, infections and infusion-related reactions.

**Conclusions:**

CT-P13 and RP have highly comparable efficacy (including PROs) and PK up to week 54. Over a 1-year period, CT-P13 was well tolerated and displayed a safety profile comparable to RP; no differences in immunogenicity were observed.

**Trial registration:**

ClinicalTrials.gov identifier: NCT01220518. Registered 4 October 2010.

**Electronic supplementary material:**

The online version of this article (doi:10.1186/s13075-016-0930-4) contains supplementary material, which is available to authorized users.

## Background

Infliximab is a human-murine chimeric monoclonal antibody directed against tumor necrosis factor (TNF). Originator infliximab (Remicade®; hereafter referred to as the reference product (RP)) was the first TNF antagonist proven to be efficacious in patients with ankylosing spondylitis (AS) [1, 2], and is now regarded as an important component of AS care [3, 4]. However, the costs of infliximab RP and other originator biologics are often high, placing considerable financial burden on healthcare systems and, in many countries, restricting patient access [5, 6]. As a result, the development of biosimilar drugs has garnered considerable interest as many originator biologics have reached or are close to patent expiry. A biosimilar may be defined as ‘a biotherapeutic product that is similar in terms of quality, safety and efficacy to an already licensed reference biotherapeutic product’ [7]. Due to their assumed lower price, biosimilars have the potential to reduce costs and increase patient access and drug therapy adherence.

CT-P13 (Remsima®, Inflectra®) is a biosimilar of RP produced in the same murine hybridoma cell line as RP and approved in Europe and elsewhere for the same indications. CT-P13 and RP are identical in amino acid sequence, pharmaceutical form, strength, composition, and route of administration [8, 9]. Secondary and tertiary structures of CT-P13 and RP are highly comparable to each other, although not identical. Both drugs bind to known infliximab ligands and receptors (e.g., TNF, Fcγ and C1q) with similar affinity, while neither bind to lymphotoxin. In vitro studies have shown that CT-P13 and RP possess equivalent TNF neutralization activity, apoptotic activity, complement-dependent cytotoxicity and antibody-dependent cellular toxicity [8, 9].

Biosimilar guidelines in Europe and the US state that demonstration of clinical comparability between a biosimilar and its innovator requires completion of comparator clinical trials assessing pharmacokinetics (PK), efficacy and safety [10, 11]. Efficacy equivalence of CT-P13 and RP was established in the PLANETRA (Programme evaLuating the Autoimmune disease iNvEstigational drug cT-p13 in Rheumatoid Arthritis) study [12]. Another study called PLANETAS (Programme evaLuating the Autoimmune disease iNvEstigational drug cT-p13 in AS patients) was conducted to assess PK equivalence and comparability of efficacy and safety in patients with AS. The primary analysis of PLANETAS was performed at week 30 of the study [13]. That analysis showed that the primary endpoint of the study—PK equivalence of CT-P13 and RP as assessed by steady state area under the serum concentration curve (AUC) and maximum serum concentration (C_max_) between weeks 22 and 30—was met. Efficacy and safety findings up to week 30 were also similar in the patients with AS.

In order to compare the extended efficacy, immunogenicity, PK and safety of CT-P13 and RP in patients with AS, PLANETAS continued up to 54 weeks (plus a subsequent off-dose period of 8 weeks). Here, we report the results of the study at week 54.

## Methods

Study methods have been reported in detail previously [13].

### Patients

AS patients aged 18–75 years meeting the 1984 modified New York classification criteria [14] for ≥3 months prior to screening, with Bath Ankylosing Spondylitis Disease Activity Index (BASDAI) score of ≥4 (range 0–10) and a visual analogue scale score for spinal pain of ≥4 (range 0–10) were eligible for the PLANETAS study. Patients were permitted to receive both oral glucocorticoids (equivalent to ≤10 mg daily prednisolone) and nonsteroidal anti-inflammatory drugs, if they had received a stable dose for ≥4 weeks prior to screening.

### Study design and treatment

This randomized, double-blind, parallel-group study (ClinicalTrials.gov identifier: NCT01220518) was conducted in 46 centers in 10 countries. Patients were randomly assigned in a 1:1 ratio to receive CT-P13 (CELLTRION Inc, Incheon, Republic of Korea) or RP (Janssen Biotech Inc, Horsham, Pennsylvania, USA), at a dose of 5 mg/kg via 2-hour intravenous infusion at weeks 0, 2 and 6, and then every 8 weeks thereafter up to week 54. Antihistamines were provided prior to infusion at the investigator’s discretion. The overall randomization code was broken for regulatory reporting purposes once the database up to week 30 had been finalized for analysis [13]. However, the study remained blinded to the investigators and patients up to week 54.

The study was carried out according to the principles of the Declaration of Helsinki and International Conference on Harmonisation good clinical practice guidelines. The study was approved by the relevant independent ethics committees (see Acknowledgements). All patients provided written informed consent.

### Study endpoints and assessments

Efficacy was assessed at baseline and weeks 14, 30 and 54. Efficacy endpoints included: patient-reported outcomes (PROs), including BASDAI, the Bath Ankylosing Spondylitis Functional Index (BASFI), and the Short Form (36) Health Survey (SF-36); the proportion of patients achieving clinical response according to Assessment of Spondyloarthritis international Society (ASAS)20 and ASAS40 criteria; the proportion of patients with ASAS partial remission; changes from baseline in Ankylosing Spondylitis Disease Activity Score (ASDAS)-C-reactive protein (CRP), Bath Ankylosing Spondylitis Metrology Index (BASMI) and chest expansion.

The proportion of patients who tested positive for anti-drug antibodies (ADAs) was assessed at baseline and weeks 14, 30 and 54 using an electrochemiluminescent immunoassay method, as previously reported [13]. The neutralizing activity of ADAs was also assessed by a flow-through immunoassay method utilizing the Gyros Immunoassay platform (Gyros AB, Sweden). Primary PK endpoints included the area under the concentration time curve over the dosing interval, at steady state between week 22 and week 30 (AUC_τ_) and the observed maximum serum concentration at steady state between week 22 and week 30 (C_max,ss_). Secondary assessments included C_max_ and minimum concentration (C_min_) immediately before the next infusion up to week 54. Blood samples were collected immediately before each treatment, at the end of each infusion, and 60 minutes post-infusion. PK analyses were performed using a flow-through immunoassay platform (Gyros AB, Sweden). The influence of ADA titer level on primary PK endpoints was assessed with a low, medium and high ADA titer based on tertile grouping of the data.

Regarding safety, adverse events (AEs) were assessed from enrollment and up to 8 weeks after last study treatment. A treatment-emergent AE (TEAE) was defined as any event not present before exposure to study treatment or an event that worsened in intensity or frequency after exposure to study treatment. Other safety assessments included vital signs monitoring, physical examination, clinical laboratory analyses, and tuberculosis (TB) monitoring. An AE of latent TB was reported if a patient had a positive result on interferon-γ release assay after a negative result at baseline together with a negative result on chest X-ray examination. Patients were monitored for infusion-related reactions, including hypersensitivity and anaphylactic reaction.

In a series of subgroup analyses, the impact of ADA status on efficacy, PK and safety was determined. For subanalysis of ASAS20 response, ADA status was determined at week 54. For C_max_ subanalysis, a non-visit-based approach was adopted to evaluate the incidence of patients who developed ADAs up to and including week 54. In subgroup analysis of infusion-related reactions, patients were grouped according to their seroconversion status, which was defined as positive if the patient had a negative ADA test followed by a positive ADA test at a subsequent visit; all other patients who provided at least one ADA test result were included in the non-seroconverted group.

### Statistical analyses

Statistical analyses were performed using Version 9.1.3 of SAS software (SAS Institute, Inc, Cary, North Carolina, USA). All efficacy analyses were performed by intention-to-treat, using the ‘missing equals excluded’ (MEX) approach. The proportion of patients achieving clinical response (ASAS20/ASAS40) was analyzed by a logistic regression model, with treatment group as a fixed effect and the stratification factors (region and baseline BASDAI score) as covariates. Treatment effect was estimated by calculating the odds ratio (OR) and 95 % confidence interval (CI). Other efficacy endpoints were analyzed using descriptive statistics.

For PK analyses, serum drug concentrations were summarized using quantitative descriptive statistics by actual treatment group, visit and time point. The PK population included patients who received at least the first five doses of study treatment, had sufficient blood concentration data to facilitate the calculation of PK parameters, and had no major protocol deviations. The safety population included all patients who received at least one (full or partial) dose of CT-P13 or RP during any dosing period.

Sensitivity analyses of ASAS response data were conducted using a last observation carried forward (LOCF) approach to compare the full-set analysis (LOCF) with the complete-case analysis (MEX).

## Results

### Patient disposition and baseline characteristics

**Fig. 1:**
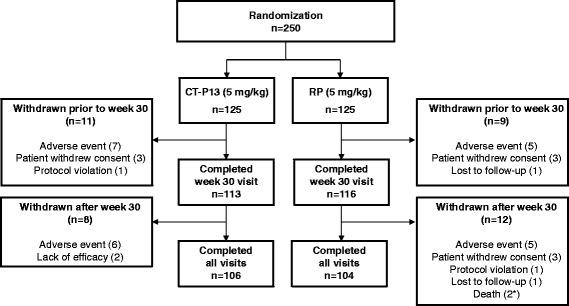
Flowchart of patient disposition. A total of 250 eligible patients were randomized into the CT-P13 group (n = 125) or RP group (n = 125) to receive 5 mg/kg of CT-P13 or RP, respectively. All 250 randomly assigned patients were included in the intention-to-treat population. *One patient randomly assigned to RP received at least one dose of CT-P13 unintentionally. *RP* reference product (i.e., reference infliximab)

Overall, 250 patients were randomly assigned to study treatment (n = 125 in each group; Fig. 1). A total of 210 patients completed 54 weeks of treatment (n = 106 (84.8 %) and 104 (83.2 %) in the CT-P13 and RP groups, respectively). The most common reason for discontinuation was AEs (n = 13 (10.4 %) and 10 (8.0 %), respectively) in each group.

Baseline patient demographics and disease characteristics are shown in Table 1. In both the CT-P13 and RP groups the majority of patients were male (n = 99 (79.2 %) and n = 103 (82.4 %), respectively), and median age was 38 years in both groups (range 18–69 years). Baseline scores for efficacy assessments, including PROs, were similar between groups.

**Table 1:** Baseline patient demographics and disease characteristics

Characteristic	CT-P13	RP	Total (n = 250)
	5 mg/kg (n = 125)	5 mg/kg (n = 125)	
Age, years	38.0 (18–69)	38.0 (18–66)	38.0 (18–69)
Gender, no. (%)			
Male	99 (79.2)	103 (82.4)	202 (80.8)
Female	26 (20.8)	22 (17.6)	48 (19.2)
Ethnicity, no. (%)			
Caucasian	97 (77.6)	92 (73.6)	189 (75.6)
Asian	16 (12.8)	13 (10.4)	29 (11.6)
Other	12 (9.6)	20 (16.0)	32 (12.8)
Height, cm	172.0 (148–198)	171.0 (147–193)	172.0 (147–198)
Weight, kg	72.70 (45.0–120.0)	76.00 (45.5–122.7)	73.75 (45.0–122.7)
Body mass index, kg/m^2^	24.39 (18.0–38.7)	25.64 (17.5–42.0)	25.12 (17.5–42.0)
ASDAS-CRP, mean ± SD	3.8 ± 0.8	3.9 ± 1.1	3.9 ± 1.0
BASDAI (stratification factor), no. (%)			
4 ~ ≤8	92 (73.6)	95 (76.0)	187 (74.8)
>8–10	33 (26.4)	30 (24.0)	63 (25.2)
BASDAI score	6.8 (3.4–10.0)	6.6 (1.8–10.0)	6.7 (1.8–10.0)
BASFI score	6.3 (0.7–9.8)	6.3 (0.1–10.0)	6.3 (0.1–10.0)
BASMI score	4.0 (0.0–9.0)	4.0 (0.0–9.0)	4.0 (0.0–9.0)
Chest expansion, cm	3.0 (0.5–9.0)	2.5 (0.0–7.0)	3.0 (0.0–9.0)
SF-36 summary scores			
Physical component	34.1 (16.2–49.7)	33.1 (15.3–54.3)	33.4 (15.3–54.3)
Mental component	38.2 (15.1–63.7)	37.2 (12.5–63.6)	37.8 (12.5–63.7)
CRP, mg/dL	1.1 (0.0–13.0)	1.4 (0.0–17.4)	1.3 (0.0–17.4)
ESR, mm/h	33.0 (2.0–110.0)	34.0 (1.0–119.0)	34.0 (1.0–119.0)

Except where indicated otherwise, values are expressed as median (minimum–maximum). *ASDAS* Ankylosing Spondylitis Disease Activity Score, *BASDAI* Bath Ankylosing Spondylitis Disease Activity Index, *BASFI* Bath Ankylosing Spondylitis Functional Index, *BASMI* Bath Ankylosing Spondylitis Metrology Index, *CRP* C-reactive protein, *ESR* erythrocyte sedimentation rate, *RP* reference product (i.e. reference infliximab), *SD* standard deviation, *SF-36* Short Form (36) Health Survey

### Efficacy

**Fig. 2:**
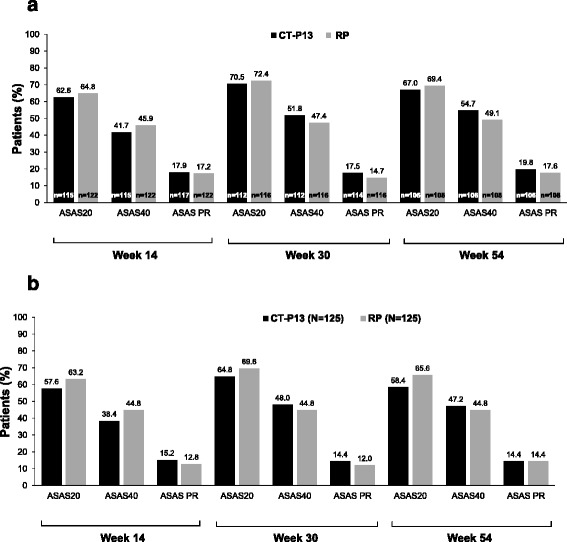
Proportion of patients with an ASAS20 response, ASAS40 response and ASAS PR* up to week 54 by treatment in the **a** ITT population (MEX approach) and **b** ITT population (LOCF approach). “n = numbers” in the bar represent the denominator of patients assessed at each time point. *PR was defined as a value of <20 on a 0–100 scale in each of the following four domains: patient global assessment, pain, function, and inflammation. *ASAS* Assessment of Spondylo Arthritis international Society (ASAS20 and ASAS40, 20 % and 40 % response according to the ASAS International Working Group criteria for improvement), *ITT* intention-to-treat, *LOCF* last observation carried forward, *MEX* missing equals excluded, *PR* partial remission, *RP* reference product (i.e. reference infliximab)

Efficacy was similar between both treatment groups, as measured by all efficacy endpoints. The proportion of patients achieving clinical response according to ASAS20 and ASAS40 criteria at weeks 14, 30 and 54 was similar between the CT-P13 and RP groups, as were ASAS partial remission rates (Fig. 2a). Logistic regression indicated no difference in ASAS20 responses between CT-P13 and RP at week 54 (0.89 (0.50, 1.59)). Similarly, there was no difference between CT-P13 and RP for ASAS40 responses at week 54 (1.26 (0.73, 2.15)). Sensitivity analyses of ASAS response rates using the full-set (LOCF) population also showed no differences between CT-P13 and RP (Fig. 2b). In a subgroup analysis performed according to ADA status, the proportion of ADA-negative patients achieving ASAS20 at week 54 was 72.3 % in the CT-P13 group and 73.1 % in the RP group. In comparison, 47.8 % and 60.0 % of ADA-positive patients in the CT-P13 and RP groups, respectively, achieved ASAS20 at week 54.

ASDAS-CRP score (mean ± standard deviation (SD)) at baseline (3.8 ± 0.8 and 3.9 ± 1.1 in the CT-P13 and RP groups, respectively) and the mean change from baseline were similar in the CT-P13 and RP groups at week 54 (−1.7 ± 1.3 and −1.7 ± 1.3).

**Fig. 3:**
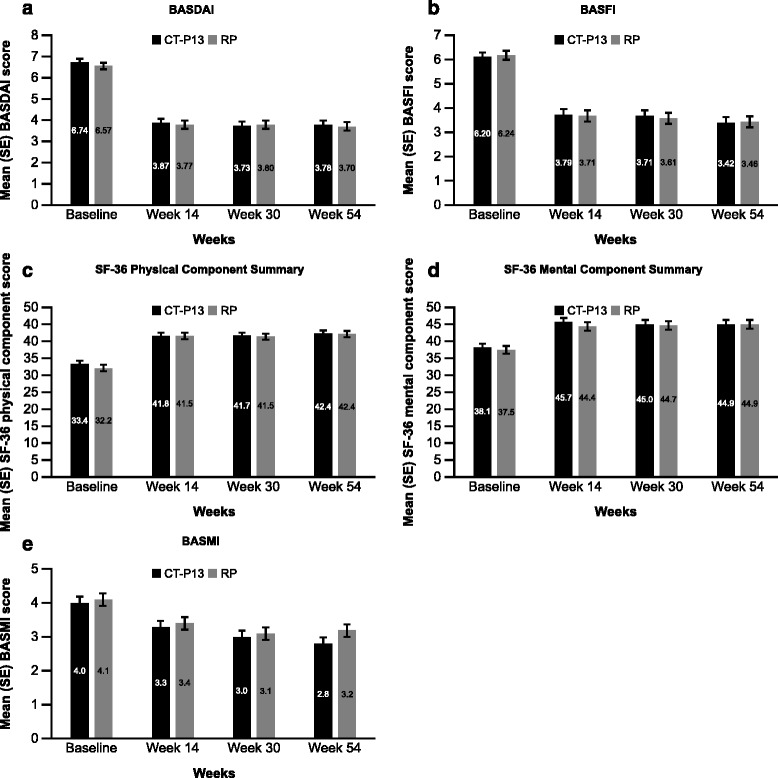
Mean **a** BASDAI, **b** BASFI, **c** SF-36 Physical Component Summary, **d** SF-36 Mental Component Summary and **e** BASMI scores up to week 54 by treatment. *BASDAI* Bath Ankylosing Spondylitis Disease Activity Index, *BASFI* Bath Ankylosing Spondylitis Functional Index, *BASMI* Bath Ankylosing Spondylitis Metrology Index, *RP* reference product (i.e. reference infliximab), *SF-36* Short Form (36) Health Survey, *SE* standard error

In terms of PROs, baseline scores of BASDAI and BASFI were similar between groups, and the scores decreased from baseline up to week 54 to a similar extent in both the CT-P13 and RP groups (mean ± SD BASDAI scores at baseline: 6.7 ± 1.4 versus 6.6 ± 1.6 in the CT-P13 and RP groups, respectively; change from baseline BASDAI scores: −2.9 ± 2.2 versus −2.8 ± 2.1 (week 14), −3.0 ± 2.2 versus −2.7 ± 2.2 (week 30) and −3.1 ± 2.3 versus −2.8 ± 2.2 (week 54); BASFI scores at baseline: 6.2 ± 1.9 versus 6.2 ± 2.2; change from baseline BASFI scores: −2.5 ± 2.1 versus −2.5 ± 2.2 (week 14), −2.6 ± 2.2 versus −2.5 ± 2.2 (week 30) and −2.9 ± 2.3 versus −2.7 ± 2.1 (week 54)) (Fig. 3a and b). Regarding the SF-36 quality of life questionnaire, scores were similar between CT-P13 and RP groups for all components of the questionnaire at baseline and weeks 14, 30 and 54. Mean SF-36 score increased from baseline to week 54 in both groups. For the physical component summary (Fig. 3c), the mean ± SD scores at baseline were 33.4 ± 6.7 and 32.2 ± 6.8, and at week 54 were 42.4 ± 8.6 and 42.2 ± 9.0 with CT-P13 and RP, respectively. For the mental component summary (Fig. 3d), the mean scores at baseline were 38.1 ± 10.3 and 37.5 ± 11.2, and at week 54 were 44.9 ± 10.9 and 44.9 ± 11.0 with CT-P13 and RP, respectively. In the CT-P13 and RP groups, respectively, mean change from baseline to week 54 was 9.26 and 10.13 for the physical component summary and 7.30 and 6.54 for the mental component summary.

Mean ± SD BASMI scores and chest expansion (cm) at baseline were 4.0 ± 2.1 versus 4.1 ± 2.1, and 3.2 ± 1.3 versus 2.9 ± 1.3 in the CT-P13 and RP groups, respectively. Mean change from baseline values were similar in the CT-P13 and RP groups (week 14: −0.7 ± 1.2 versus −0.7 ± 1.4; week 30: −1.0 ± 1.4 versus −0.9 ± 1.4; week 54: −1.1 ± 1.5 versus −0.9 ± 1.6 for BASMI (Fig. 3e); week 14: 0.4 ± 1.0 versus 0.7 ± 1.0; week 30: 0.6 ± 1.4 versus 0.8 ± 1.2; week 54: 0.7 ± 1.4 versus 0.9 ± 1.1 for chest expansion).

### Immunogenicity

The proportion of patients with ADAs was similar between the CT-P13 and RP groups at each time point (week 14: n = 11 (8.6 %) and n = 13 (10.7 %); week 30: n = 32 (25.0 %) and n = 25 (20.5 %); week 54: n = 25 (19.5 %) and n = 28 (23.0 %)). The vast majority of patients who were ADA-positive also had neutralizing activity in both the CT-13 and RP groups (week 14: n = 10 and n = 13; week 30: n = 31 and n = 24; week 54: n = 25 and n = 28).

### Pharmacokinetics

**Fig. 4:**
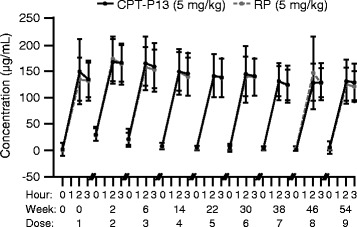
Mean (SD) serum PK parameters of CT-P13 and RP (PK population). Note: values below the LLOQ have been set equal to the LLOQ. *LLOQ* lower limit of quantification, *PK* pharmacokinetics, *RP* reference product (i.e. reference infliximab), *SD* standard deviation

A total of 223 (89.2 %) patients were included in the PK population (n = 113 and n = 110 in the CT-P13 and RP groups, respectively). C_max_ and C_min_ values were similar between the two treatment groups up to week 54 (Fig. 4).

**Fig. 5:**
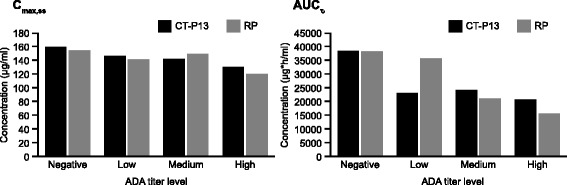
Primary PK parameters (C_max,ss_ and AUC_τ_) by ADA titer level at week 30 (PK population). Note: titer levels were defined as low, medium and high titer based on tertile grouping of the data. *ADA* anti-drug antibodies, *AUC*_*τ*_ area under the concentration time curve over the dosing interval, at steady state between week 22 and week 30, *C*_*max,ss*_ the observed maximum serum concentration at steady state between week 22 and week 30, *PK* pharmacokinetics, *RP* reference product (i.e. reference infliximab)

The influence of ADAs on primary PK endpoints (C_max,ss_ and AUC_τ_) was also investigated. Figure 5 shows these endpoints by ADA titer level at week 30 in the CT-P13 and RP groups. There was a trend for both C_max,ss_ and AUC_τ_ to be lower with higher ADA titer levels. This impact of ADAs on drug exposure was similar in the two treatment groups. In a related subgroup analysis, mean (coefficient of variation) C_max_ was comparable in the CT-P13 and RP groups at week 54 in both ADA-negative (142.98 (27.6) versus 135.27 (22.6) μg/mL, respectively) and ADA-positive (122.53 (31.1) versus 117.16 (25.6) μg/mL) patients.

### Safety

Due to incorrect kits being dispensed, three patients randomly assigned to the RP treatment group received one dose of CT-P13 in this study. Therefore, the safety population comprised 128 and 122 patients in the CT-P13 and RP groups, respectively.

The proportion of patients who experienced at least one TEAE was similar in the CT-P13 and RP groups (n = 95 (74.2 %) and 82 (67.2 %), respectively). Most TEAEs were mild or moderate in intensity. TEAEs considered by the investigator to be related to study treatment were reported in 64 (50.0 %) and 63 (51.6 %) patients, respectively. The most common of these events are shown in Table 2. Related TEAEs occurring in >4 % of patients were (for CT-P13) abnormal liver function test (n = 16 (12.5 %)), upper respiratory tract infection (n = 12 (9.4 %)), infusion-related reaction (n = 11 (8.6 %)) and latent TB (9 (7.0 %)), and (for RP) abnormal liver function test (n = 15 (12.3 %)), infusion-related reaction (n = 15 (12.3 %)), upper respiratory tract infection (n = 8 (6.6 %)) and latent TB (6 (4.9 %)). Infusion-related reactions affected more ADA-positive than ADA-negative patients in both treatment groups (CT-P13: 6/44 (13.6 %) versus 5/84 (6.0 %); RP: 11/39 (28.2 %) versus 4/83 (4.8 %)).

**Table 2:** Treatment-related adverse events reported in at least 1 % of all (total) patients, n (%)

	CT-P13	RP	Total
	(n = 128)	(n = 122)	CT-P13 + RP (n = 250)
Abnormal liver function test	16 (12.5)	15 (12.3)	31 (12.4)
Infusion-related reaction	11 (8.6)	15 (12.3)	26 (10.4)
Upper respiratory tract infection	12 (9.4)	8 (6.6)	20 (8.0)
Latent tuberculosis	9 (7.0)	6 (4.9)	15 (6.0)
Urinary tract infection	5 (3.9)	4 (3.3)	9 (3.6)
Neutropenia	4 (3.1)	3 (2.5)	7 (2.8)
Elevated serum creatine phosphokinase	4 (3.1)	2 (1.6)	6 (2.4)
Rash	2 (1.6)	4 (3.3)	6 (2.4)
Headache	3 (2.3)	1 (0.8)	4 (1.6)
Herpes virus infection	1 (0.8)	3 (2.5)	4 (1.6)
Sinusitis	2 (1.6)	1 (0.8)	3 (1.2)
Tuberculosis	2 (1.6)	1 (0.8)	3 (1.2)

*RP* reference product (i.e. reference infliximab)

Two cases of active TB were reported in the CT-P13 group and one case was reported in the RP group. A single case of malignancy, which was considered to be unrelated to treatment, was reported in the CT-P13 group (basal cell carcinoma). The onset date of this event was unknown, but was believed to have started 2 years previously, based on the medical history of the patient (melanocyte nevus). Treatment for the event was excision of a skin nodule of the nose. The event was considered to have recovered/resolved according to the investigator.

The proportion of patients who experienced at least one serious AE (SAE) was similar between the CT-P13 and RP groups (n = 10 (7.8 %) and 8 (6.6 %), respectively), as was the proportion who had an SAE considered related to treatment (n = 4 (3.1 %) and 5 (4.1 %); see Additional file 1 for details of treatment-related SAEs). The number of patients with at least one TEAE leading to discontinuation was comparable between the CT-P13 and RP groups (n = 11 (8.6 %) and 9 (7.4 %), respectively). Of these, 9 (7.0 %) and 7 (5.7 %) were considered to be related to CT-P13 and RP, respectively. Overall, treatment-related events leading to discontinuation of more than one patient were TB in two patients from the CT-P13 group and one in the RP group, two abnormal liver function tests (one in each group), and infusion-related reactions in one patient from the CT-P13 group and five from the RP group. Two deaths occurred during the study, one in each treatment group. Both were due to car accidents and were considered not related to study treatment.

## Discussion

PLANETAS was a multinational, randomized, double-blind biosimilar study that evaluated the equivalence of PK and comparability of efficacy and safety between CT-P13 and RP up to 54 weeks in patients with active AS.

Similar clinical efficacy was observed between CT-P13 and RP up to week 54. ASAS responses at weeks 30 and 54, respectively, were comparable in the two treatment groups. PROs—including mean BASDAI, BASFI and SF-36 scores—and other efficacy endpoints such as BASMI score and chest expansion were also similar in both treatment groups up to week 54. The similarity in PROs is notable because, unlike the other efficacy endpoints employed in the study, these are patient-led evaluations.

Thirty-week efficacy data in PLANETAS were comparable to 24-week data from a pivotal placebo-controlled clinical study of RP in AS (ASSERT) [2, 13]. Analogously, the efficacy data seen here for CT-P13 at week 54 are in line with findings previously reported for RP in different clinical trials with a similar duration [15–17]. For example, in the current analysis, the ASAS20 responses and improvement of BASDAI and BASFI scores with CT-P13 at 54 weeks were 67 %, 3.1 and 2.9, respectively. Similar improvements in these measurements were also observed at weeks 48–58 in historical studies with RP (ASAS20 at week 58 = 75 %; BASDAI from 6.6 at week 0 to 2.4 at week 54; BASFI from 6.1 at baseline to 4.4 at week 48) [15–17]. While there are inherent limitations associated with comparing across trials due to differences in patient populations and methods, it is reassuring that the data for CT-P13 are generally in line with historical observations for RP. Long-term efficacy equivalence of CT-P13 and RP has also been established in a 54-week analysis of the PLANETRA study in rheumatoid arthritis (RA). An American College of Rheumatology (ACR)20 response was achieved in a highly similar proportion of patients treated with CT-P13 and RP [18].

The long-term PK profiles (C_max_ and C_min_) of CT-P13 and RP were similar to each other in the current study through to week 54. The C_max_ data reported here are consistent with PLANETAS data reported at 30 weeks and those of studies adopting a similar RP dosing pattern in Crohn’s disease [13, 19]. Similarity of PK was also observed between CT-P13 and RP in patients with RA in the PLANETRA study [12, 18].

CT-P13 was well tolerated up to week 54, with a safety profile comparable to that of RP. As previously reported at week 30 for PLANETAS [13], there was no notable difference between study arms in the incidence of AEs, SAEs, infections and infusion-related reactions; this trend continued up to week 54. The safety profile observed with CT-P13 in the current study is also generally aligned with the safety profile of RP in historical studies involving patients with AS and RA [1, 2, 15–17, 20–29]. One treatment-unrelated case of basal cell carcinoma was reported with CT-P13. It is believed that the malignancy started 2 years prior to study participation. Following appropriate treatment (excision), the event was considered to have resolved.

It is well documented that development of ADAs against infliximab is associated with decreased clinical response and drug serum concentration [13, 30–32]. We also observed lower ASAS20 response rates and lower C_max_ values at week 54, as well as a higher incidence of infusion-related reactions in ADA-positive compared with ADA-negative patients. These ADA effects were comparable in both the CT-P13 and RP groups. Further analysis of PK parameters with respect to ADA levels found a trend for both AUC_τ_, and C_max,ss_ to be lower with increasing ADA titer level, although a formal statistical inference was not made due to a limited statistical power.

Further extensions of the current study and the PLANETRA study were conducted to examine the efficacy and safety of switching treatment from RP to CT-P13 in patients with AS and RA, respectively. Additionally, a formal randomized double-blind ‘switching’ clinical trial is currently progressing in Norway. This trial (‘NOR-SWITCH’; ClinicalTrials.gov identifier: NCT02148640) is comparing the safety and efficacy of switching from RP to CT-P13 versus continued treatment with RP in several indications, including RA. Data from the PLANETAS/PLANETRA extensions and NOR-SWITCH will provide further evidence on the ability to switch from RP to CT-P13.

The main limitation of this analysis is that PLANETAS was primarily designed to compare the PK profiles of CT-P13 and RP at 30 weeks. While prospectively planned, the longer-term efficacy, safety and PK data presented here are from a secondary analysis of the study.

## Conclusions

This multinational, randomized, biosimilar study in patients with active AS demonstrated that CT-P13 and RP have highly comparable efficacy, safety, immunogenicity and PK profiles up to week 54. Together with the findings from the PLANETRA study in patients with RA, these data further demonstrate the similarity between CT-P13 and RP in diverse clinical settings.

## Additional file

Additional file 1:
**Overview of treatment-related SAEs occurring by severity, n (%).** (DOC 31 kb)

## Abbreviations

ADA: Anti-drug antibody
AE: Adverse event
AS: Ankylosing spondylitis
ASAS: Assessment of Spondylo Arthritis international Society
ASDAS: Ankylosing Spondylitis Disease Activity Score
AUC: Area under the curve
AUC_τ_: Area under the concentration time curve over the dosing interval, at steady state between week 22 and week 30
BASDAI: Bath Ankylosing Spondylitis Disease Activity Index
BASFI: Bath Ankylosing Spondylitis Functional Index
BASMI: Bath Ankylosing Spondylitis Metrology Index
CI: Confidence interval
C_max_: Maximum serum concentration
C_max,ss_: Maximum serum concentration at steady state between week 22 and week 30
C_min_: Minimum serum concentration
CRP: C-reactive protein
LOCF: Last observation carried forward
MEX: Missing equals excluded
OR: Odds ratio
PK: Pharmacokinetics
PRO: Patient-reported outcome
RA: Rheumatoid arthritis
RP: Reference product
SAE: Serious adverse event
SD: Standard deviation
SF-36: Short Form (36) Health Survey
TB: Tuberculosis
TEAE: Treatment-emergent adverse event
TNF: Tumor necrosis factor
