# Supplementary material for: Comparable long-term efficacy, as assessed by patient-reported outcomes, safety and pharmacokinetics, of CT-P13 and reference infliximab in patients with ankylosing spondylitis: 54-week results from the randomized, parallel-group PLANETAS study
Source: Arthritis Res Ther. 2016 Jan 20;18:25. doi: 10.1186/s13075-016-0930-4 (PMC4721187; doi:10.1186/s13075-016-0930-4)
Supplement: Additional file 1: — Overview of treatment-related SAEs occurring by severity, n (%). (DOC 31 kb) [file 13075_2016_930_MOESM1_ESM.doc]

Additional File 1: Overview of treatment-related SAEs occurring by severity, n (%).

|  | **CT-P13**  **5 mg/kg (n=128)** | | | **RP  5 mg/kg (n=122)** | | |
| --- | --- | --- | --- | --- | --- | --- |
| **n (%)** | **Mild** | **Moderate** | **Severe** | **Mild** | **Moderate** | **Severe** |
| Tuberculosis | **-** | 2 (1.6)^*^ | - | **-** | - | 1 (0.8) |
| Cellulitis | **-** | **-** | **-** | 1 (0.8)^*^ | **-** | **-** |
| Wound infection | **-** | **-** | **-** | 1 (0.8)^*^ | **-** | **-** |
| Infusion-related reaction | **-** | 1 (0.8) | **-** | - | 2 (1.6) | 1 (0.8) |
| Esophageal perforation | **-** | - | 1 (0.8)^*^ | **-** | - | - |
| Demyelination | 1 (0.8) | **-** | **-** | **-** | **-** | **-** |

Note: The event was considered to be related if the relationship was defined as “possible,” “probable” or “definite.”
* There were two patients (1 in the CT-P13 group, 1 in the RP group) with two treatment-related SAEs reported.
RP, reference product (i.e. reference infliximab); SAE, serious adverse event.
